# Magnitude of enteropathogens and associated factors among apparently healthy food handlers at Wolkite University Student’s Cafeteria, Southern Ethiopia

**DOI:** 10.1186/s13104-019-4599-z

**Published:** 2019-09-11

**Authors:** Temesgen Abera Bafa, Ebrahim Muktar Sherif, Admasu Haile Hantalo, Gashaw Garedew Woldeamanuel

**Affiliations:** 1 Department of Medical Laboratory Sciences, College of Medicine and Health Sciences, Wolkite University, P.O. Box 07, Wolkite, Ethiopia; 2 Department of Public Health, College of Medicine and Health Sciences, Wolkite University, Wolkite, Ethiopia; 3 Department of Biomedical Sciences, School of Medicine, College of Medicine and Health Sciences, Wolkite University, Wolkite, Ethiopia

**Keywords:** Food handlers, Intestinal parasites, Salmonella, Shigella

## Abstract

**Objective:**

The study aimed to assess enteropathogens carriage rate and risk factors among apparently healthy food handlers at Wolkite University cafeteria, Southern Ethiopia.

**Results:**

Cross-sectional study was conducted among 170 food handlers to collect socio-demographic and related data by using structured questionnaires. Stool samples were collected and subjected to microscopic examination and cultured to determine intestinal parasites. Among the total study participants, 66% of them were found to be carriers of enteropathogens: bacteria (*Salmonella typhi*, *Shigella* species), and intestinal parasites (*Ascaris lumbricoides*,*Taenia species, Giardia lamblia*, *Entameoba histolytica/dispar*, *Enterobius vermicularis*, *Hook worm* and *Trichuris trichiura)*. All *Salmonella* and *Shigella* isolates were sensitive to ceftriaxone, ciprofloxacin, and gentamicin. A significant association was found between hand washing practice before food preparation and isolated pathogens [p = 0.013]. A significant proportion of food handlers were found to be carriers of pathogens which require a periodic screening and antibiotic therapy monitoring.

## Introduction

Human’s health and well-being is indispensably related on safe delivery, sound and nutritious food [1]. However, sometimes, health problem can be fronted by consumption of contaminated foods resulted in food-borne disease. Moreover, diseases spread through food are common and persistent problems that result in appreciable morbidity and mortality across the globe especially to developing countries [2, 3].

The World Health Organization (WHO) estimated that up to 70% of the population suffer from diarrheal diseases in developing countries, with an estimated 2 million deaths per year [4, 5]. The diseases are caused mostly by food-borne microbial pathogens like Salmonella, Campylobacter, pathogenic Escherichia coli, Shigella, and other related intestinal parasites [6, 7]. These organisms may exist on food handler’s skin, from which they may be transmitted to cooked moist protein-rich foods, and become food contaminants agents if these foods are then kept for several hours without refrigeration [8–11]. Fingernails compared to other parts of the hand, harbors many microorganisms and is difficult to clean [10].

*Salmonella typhi* is one of the major causes of food and water borne gastroenteritis in humans [12–14] and remains an important health problem worldwide of which 16 million new cases and 600,000 deaths of typhoid fever annually [15] with antimicrobial resistance emergence as a current concern [16]. Evidences showed that different predisposing factors like food handlers’ personal hygiene, knowledge and practice of food hygiene are the major determinants for food contamination and outbreaks of food poisoning in developing countries including Ethiopia where there is a poor regulatory system for food hygiene and inadequate food safety laws [17].

Many developing countries including Ethiopia and Nigeria still emphasize medical examination as a pre-requisite to work as a food handlers [18]. In addition, recent studies conducted in Arba Minch, Southern Ethiopia and Gondar, Northern Ethiopia showed that food handlers who had medical checkup were more likely to have good food handling practice as compared to those who had no medical checkup [19, 20]. Similarly, food safety training is one method for combating food safety risks [21]. Researchers have confirmed that adequate food safety training of all employees can have a positive impact on health inspection scores and on some food safety behaviors, such as hand washing, in the retail food industry [22]. Many food safety professionals have agreed that employee training and implementation are essential in preventing food-borne illness [21, 22]. Furthermore, other studies [23–25] indicated that food handlers who attended food safety training were more likely to have good practice towards food sanitations.

Food safety issues are common public health issues in our setups which are not well understood and have received little attention. Meals served at establishments in higher learning institution are prepared in large quantities pave a way to contamination, and there is a greater potential for the occurrence of food borne disease outbreaks if basic sanitary practices are not properly maintained [26]. Thus, this study aimed at determining the prevalence of intestinal pathogens and assessing the hygienic practices among food handlers at Wolkite University cafeteria, Southern Ethiopia.

## Main text

### Methods

#### Study design and period

A cross sectional study was conducted among food handlers at Wolkite University cafeteria, Southern Ethiopia from December, 2016 to February, 2017. At that time, the cafeteria had 340 food handling personnel (301 females and 39 males) serving for about 11,000 students.

#### Sampling size determination and sampling techniques

Sample size was determined using single population proportion formula taking the prevalence of Salmonella species as 6.9% from previous study [19], confidence level of 95%, 4% margin of error and with the assumption of 10% non response rate. Thus, the final sample size became 170.

All food handlers from the University food handling establishments were considered and divided into four groups based on their job description as cookers, servers, cleaners and choppers. Then, the total sample size was allocated to each group based on probability proportional to size sampling technique. Accordingly, 68 cookers, 49 servers, 30 cleaners and 23 choppers were selected by using simple random sampling method. Food handlers who were symptomatic, those who had taken antibiotics, anti-helminthes within 3 weeks prior to the study and newly recruited food handlers were excluded from the current study.

#### Data collection procedures

Personal data, hygienic profile, knowledge and attitude assessments were collected by face to face interview using structured questionnaire adopted from similar survey and literatures.

*Ova/parasite detection* A stool sample was collected from each food handler using sterile stool cup and a loop full used for wet mount. Intestinal parasites were examined and identified by direct microscopic examination of wet stool preparations, with a small amount of the respective stool sample emulsified in a drop of physiological saline, iodine solution and formol-ether concentration sedimentation techniques as per the standards. The parasites identified in any one of the three techniques from a single specimen were reported as positive [18].

*Culturing of Salmonella and Shigella species* Stool samples were cultured in appropriate culture media (Oxoid, UK) for bacteriological investigations. Stool specimens were pre-enriched with Selenite F broth and inoculated to Xylose Lysine Deoxycholate (XLD) by incubating at 37 °C for 24 h for isolation of *Shigella* and *Salmonella* isolates. Colony characteristics and biochemical tests were applied to differentiate entero-pathogens, glucose and lactose fermentation, hydrogen sulfide production, Kliger iron agar, indole, Simon’s citrate agar, lysine iron agar, urea, and motility [21]. To differentiate from other enterobacterceae and as a confirmatory test, we have used API-20E (Biomerieux, France).

Antibiotic susceptibility was performed by Kirby–Bauer disc diffusion on Muller-Hinton agar using Norfloxacillin (10 μg), Gentamicin (10 μg), Ceftriaxone (30 μg), Ciprofloxacin (5 μg), Tetracycline (30 μg), Chloramphenicol (30 µg) and Trimethoprim-Sulphamethoxazole (25 μg). The reading and interpretation of the results as sensitive, intermediate, and resistant were conducted in reference to CLSI, 2015 [22].

*Quality control* Standard operating procedures (SOPs) were strictly followed during laboratory specimen’s collection, processing and culturing. American type culture collection bacterial reference strain of *Escherichia coli* ATCC25922 was used as a quality control for antibiotic susceptibility testing.

#### Data analysis

The data were edited, coded, and entered into SPSS version 20 statistical software. Descriptive statistics were used to determine frequencies and percentages. The relationship between variables was computed using Chi square and *p* value less than 0.05 was considered as statistically significant.

### Results

#### Socio-demographic characteristics

The study encompassed 170 food handlers, of which 149 were females (149/170, 87.6%) or (301/340, 88.5%), whose age ranged from 18 to 57 years with an average of 26.5 years. Out of the total study participants, 60.0% were married and 61.8% of them were worked for 2–3 years (Table 1).

**Table 1:** Socio-demographic characteristics and intestinal parasites positivity at Wolkite University Cafeteria food handlers from January to May, 2016 (n = 170)

Characteristics	Frequency, n (%)	Positive for parasite, n (%)
Sex		
Female	149 (87.6)	85 (86.7)
Male	21 (12.4)	13 (13.3)
Age (years)		
12–19	16 (9.4)	9 (9.2)
20–40	146 (85.9)	84 (85.7)
≥ 41	8 (4.7)	5 (5.1)
Educational levels		
Illiterate	12 (7.1)	7 (7.1)
1–6 grade	44 (25.9)	25 (25.5)
7–12 grade	113 (66.5)	65 (66.3)
> 12 grade	1 (0.6)	1 (1.0)
Service year		
< 1 year	46 (27.1)	24 (24.5)
1–2 years	68 (40.0)	39 (39.8)
> 2 years	56 (32.9)	35 (35.7)
Job type		
Cooker	68 (40.0)	41 (24.1)
Server	49 (28.8)	28.8 (17.6)
Cleaner	30 (17.7)	17.7 (4.7)
Chopper	23 (13.5)	13.5 (11.2)
Total	170 (100.00)	98 (57.6)

#### Hand washing practices and knowledge

Of all food handlers, 112 (65.9%) had the habit of hand washing after toilet, of which 37.5% washed with soap and water whereas majority, 62.5% of the participants used water only. Of the total food handlers, about 84 (49.4%) and 87 (51.2%) had a habit of hand washing after touching dirty objects and before eating food, respectively. One hundred fifty-two of the food handlers knew at least one type of food borne disease, of which 55.3% answered that food contamination as a route. About 72.0% food handlers replied unhygienic food preparation was a cause of food borne diseases followed by germs, 60% while the least, 36 (21.1%) has responded chemicals as the source of food contaminants (Additional file 1: Table S1).

#### Prevalence of intestinal parasites

**Fig. 1:**
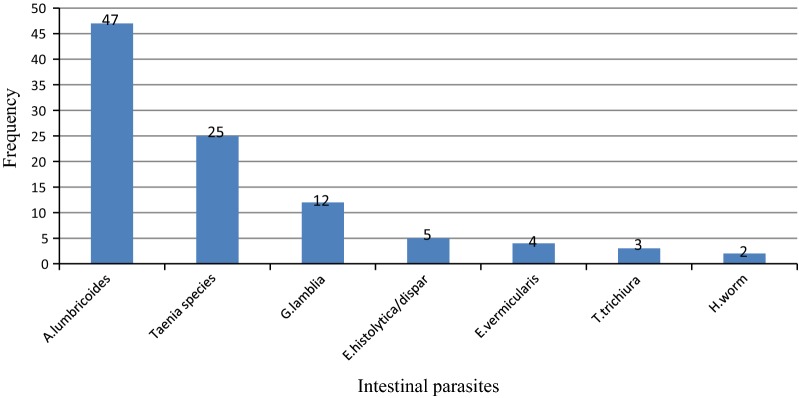
Identity, number and distribution of intestinal parasites isolated from 170 Food handlers at Wolkite University Cafeteria from January to May, 2016, Ethiopia

A total of 14 bacterial strains and 98 intestinal parasites, of which 27.6%, 14.7% and 7.1% were found to be positive for *Ascaris lumbricoides, Taenia species and Giardia lamblia*, respectively. About 5.9% of the food handlers were carriers of *S. typhi* while 32 (32.6%) had miscellaneous parasitic infections (Fig. 1 and Additional file 2: Figshare S1). Moreover, only two of the food handlers who were serving as cookers were found to be co-infected with both Salmonellae and Shigella species whilst one cooker was found to be positive for bacterial and parasitic infections. In general, these 14 bacterial strains were detected from the 12 food handlers and Chi square test was done on these 12 food handlers, of which two of them were found to be infected by both bacterial strains. Hence, to avoid doubling error, the Chi square association was done on those 12 food handlers (Additional file 1: Table S1).

#### Risk factors for Enteropathogens

We found no significant association between majority of the hygienic practice of food handlers and isolated bacterial pathogens, Salmonella and Shigella species. However, a significant association was found between hand washing practice before food preparation and isolated pathogens [p = 0.013] (Additional file 1: Table S1).

#### Antimicrobial susceptibility pattern

All *Salmonella* and *Shigella* species showed 100% resistance to both ampicillin and amoxacillin. Nevertheless, both isolates were found to be 100% sensitive to ceftriaxone, ciprofloxacillin, norfloxacillin, and gentamicine. The overall multi-drug resistance rate of Salmonella and *Shigella* species was found to be 100% and 75%, respectively (Additional file 3: Tabshare S1).

### Discussion

This study revealed that about 112 (66%) of the food handlers were found to be carriers of one or more of the pathogenic organisms of which *Ascaris lumbricoides, Taenia* species *and Giardia lamblia* were the most common and represented 55.3% of the total infections. This finding is higher than the overall prevalence of intestinal parasites among food handlers reported from other studies conducted in Gondar (29.1%) [11], Bahir Dar (41.1%) [17] and Hawassa (63%) [27]. High intestinal parasitosis in our study may be attributed to poor personal hygienic practices and poor environmental sanitation. In the present study, *A. lumbrcoides* was the most common intestinal parasite isolated in 27.6% of the food handlers. Relatively similar findings were reported from study conducted in Ethiopia (18.1%) [28, 29] and Jordan [30]. In our study, the prevalence of *G. lamblia* was 7.1%, which is inconsistent with reports on prevalence (2–5%) of the parasite in developing countries [31, 32].

The current study also depicted that 4 (2.4%) of food handlers were found to be positive for *Shigella* species, comparable with a study done in Gondar (3.1%) [33]. Disparate findings were also reported in Addis Ababa University Student’s Cafeteria [29], North India [34, 35], Sudan (1.3%) [8] and Jordan (1.4%) [30]. In this study, all Shigella isolates were found to be sensitive to ciprofloxacin and norfloxacin. However, the isolates showed high resistance for commonly used antibiotics. This finding corroborated with a study done in the University of Gondar hospital which showed high resistance to tetracycline (90%), cotrimoxazole (84.6%), ampicilin (78.9%) and chloramphenicol (67.8%) and lower resistance to gentamycin (12.2%), ciprofloxacin (2.2%) and norfloxacin (1.1%) [30].

Out of the total study subjects, majority of the food handlers had low educational status, which agrees with a study conducted in Amritsar City (94.1%) [36]. Our study result revealed that, none of the food handlers had medical checkup including stool examination in the past which is in contrary to the findings reported from Mekelle Univesity cafeteria where 63.2% of food handlers have undergone medical checkup in the last 6 months [37]. But, our finding is relatively similar with a study conducted in Hawassa town where 0.6% of the study participants had medical check-up [27]. Low tendency of recruiting food handlers without considering certificate as a basic criterion and low monthly payment for food handlers could contribute to this difference.

### Conclusions and recommendations

Significant proportion of food handlers were affected by intestinal parasites and *S. typhi* carriage rate. Significant association was observed between hand washing practice before food preparation and isolated Salmonella. The overall multi-drug resistance rate of Salmonella and *Shigella* species was found to be 100% and 75%, respectively. Thus, concerned bodies should made prompt intervention to reduce further transmission of food borne diseases, including set up periodic medical checkup, training on safe food handling and hand washing practice during critical times.

## Limitations of the study

As far as the limitations of this study concerned, there are some limitations. First, Salmonella serotype and Taenia species were not identified due to resource unavailability. Second, the smaller sample size of this study made it unable to use advanced analysis to make associations. Third, the cross sectional nature of the study had made it unable to establish the causal relation between knowledge of pregnancy danger signs and explanatory variables. Finally, we did not made any attempt to check for viral infections in food handlers. Besides, none of the food handlers were symptomatic at the time of testing.

## Supplementary information

**Additional file 1: Table S1.** Hygienic practice of food handlers in relation to Salmonella and Shigella species, working at cafeteria of Wolkite University from January to May 2016 (n = 170).

**Additional file 2: Figshare S1.** Identity, number and distribution of enteric bacteria isolated from 170 Food handlers at Wolkite University Cafeteria from January to May, 2016, Ethiopia.

**Additional file 3: Tabshare S1.** Antimicrobial susceptibility pattern of *Salmonella* and *Shigella* Species from stool sample of food handlers at Wolkite University cafeteria, January to May, 2016 (n = 170).

## Data Availability

The datasets used and/or analyzed during the current study are available from the corresponding author on reasonable request.

## Abbreviations

ANC: antinatal care
ELISA: Enzyme Linked Immuno Sorbent Assay
HBV: hepatitis B virus
HCV: hepatitis C virus
WHO: World Health Organization
