# Supplementary material for: Magnitude of enteropathogens and associated factors among apparently healthy food handlers at Wolkite University Student’s Cafeteria, Southern Ethiopia
Source: BMC Res Notes. 2019 Sep 11;12:567. doi: 10.1186/s13104-019-4599-z (PMC6737660; doi:10.1186/s13104-019-4599-z)
Supplement: Supplementary file 1 — Additional file 1: Table S1. Hygienic practice of food handlers in relation to Salmonella and Shigella species, working at cafeteria of Wolkite University from January to May 2016 (n = 170). [file 13104_2019_4599_MOESM1_ESM.docx]

Table S1: Hygienic practice of food handlers in relation to Salmonella and Shigella species, working at cafeteria of Wolkite University from January to May 2016 ( n = 170)

| **Characteristics Salmonella/Shigella χ^2^ p -value**  **Positive, n(%) Negative, n (%)** |
| --- |
| **Certified in food preparation & handling**  Yes 2 (16.7) 0 (0) NA NA  No 10 (83.3) 158(100)  **Medical Check up in last six months**  Yes 2 (16.7) 42(26.6) NA NA  No 10 (83.3) 116 (73.4)  **Hand washing practice after toilet by**  Water only 8 (66.7) 103 (65.2) NA NA  Water and soap 4 (33.3) 55 (34.8)  **Hand washing practice after touching dirty**  **objects in between handling food items**  Yes 5 (41.7) 79 (50) 0.31 0.578  No 7 (58.3) 79 (50)  **Hand washing practice before food preparation**  Yes 10 (83.3) 154 (97.5) NA NA  No 2 (16.7) 4 (2.5)  **Hand washing practice before eating food**  Yes 2 (16.7) 85 (53.8) 6.15 0.013*  No 10 (83.3) 73 (46.2)  **Hand washing after touching nose in**  **between handling food items**  Yes 4 (33.3) 81(51.3) 1.43 0.231  No 8 (66.7) 77 (48.7) |

χ^2^: chi-square; NA: not applicable; *: statistically significant
