# Supplementary material for: Magnitude of enteropathogens and associated factors among apparently healthy food handlers at Wolkite University Student’s Cafeteria, Southern Ethiopia
Source: BMC Res Notes. 2019 Sep 11;12:567. doi: 10.1186/s13104-019-4599-z (PMC6737660; doi:10.1186/s13104-019-4599-z)
Supplement: Supplementary file 2 — Additional file 2: Figshare S1. Identity, number and distribution of enteric bacteria isolated from 170 Food handlers at Wolkite University Cafeteria from January to May, 2016, Ethiopia. [file 13104_2019_4599_MOESM2_ESM.docx]

Figshare S1: Identity, number and distribution of enteric bacteria isolated from 170 Food handlers at Wolkite University Cafeteria from January to May, 2016, Ethiopia
