# Supplementary material for: Magnitude of enteropathogens and associated factors among apparently healthy food handlers at Wolkite University Student’s Cafeteria, Southern Ethiopia
Source: BMC Res Notes. 2019 Sep 11;12:567. doi: 10.1186/s13104-019-4599-z (PMC6737660; doi:10.1186/s13104-019-4599-z)
Supplement: Supplementary file 3 — Additional file 3: Tabshare S1. Antimicrobial susceptibility pattern of Salmonella and Shigella Species from stool sample of food handlers at Wolkite University cafeteria, January to May, 2016 (n = 170). [file 13104_2019_4599_MOESM3_ESM.docx]

Tabshare S1: Antimicrobial susceptibility pattern of *Salmonella* and *Shigella* Species from stool sample of food handlers at Wolkite University cafeteria, January to May, 2016 (n=170)

| **Antibiotic (Potency)** | ***Salmonella* Spp.** | | ***Shigella* Spp.** | |
| --- | --- | --- | --- | --- |
|  | S | R | S | R |
| Amoxacillin(30µg) | 0 | 10 (100) | 0 | 10 (100) |
| Ampicillin(10µg) | 0 | 10 (100) | 0 | 10 (100) |
| Ceftriaxone(30µg) | 10 (100) | 0 | 4 (100) | 0 |
| Chloroamphenicol(30µg) | 0 | 10 (100) | 1 (25) | 3 (75) |
| Ciprofloxacin(5µg) | 10 (100) | 0 | 4 (100) | 0 |
| Norfloxacin(10µg) | 10 (100) | 0 | 4 (100) | 0 |
| Gentamicin(10µg) | 10 (100) | 0 | 4 (100) | 0 |
| SXT(1.25/23.75µg) | 0 | 10 (100) | 3 (75) | 1 (75) |
| Tetracycline(30µg) | 1 (10) | 9 (90) | 3 (75) | 1 (75) |
| Data are presented as *n* (%); R:Resistance; S:Sensitive; SXT:Trimethoprim sulphamethoxazole | | | | |
